# Modification of the *Campylobacter jejuni* flagellin glycan by the product of the Cj1295 homopolymeric-tract-containing gene

**DOI:** 10.1099/mic.0.038091-0

**Published:** 2010-07

**Authors:** Paul Hitchen, Joanna Brzostek, Maria Panico, Jonathan A. Butler, Howard R. Morris, Anne Dell, Dennis Linton

**Affiliations:** 1 Division of Molecular Biosciences, Faculty of Natural Science, Imperial College, London SW7 2AY, UK; 2 Centre for Integrative Systems Biology at Imperial College, Faculty of Natural Science, Imperial College, London SW7 2AY, UK; 3 Faculty of Life Sciences, University of Manchester, Manchester M13 9PT, UK; 4 M-SCAN Ltd, Wokingham, Berkshire RG41 2TZ, UK

## Abstract

The *Campylobacter jejuni* flagellin protein is *O-*glycosylated with structural analogues of the nine-carbon sugar pseudaminic acid. The most common modifications in the *C. jejuni* 81-176 strain are the 5,7-di-*N-*acetylated derivative (Pse5Ac7Ac) and an acetamidino-substituted version (Pse5Am7Ac). Other structures detected include *O-*acetylated and *N-*acetylglutamine-substituted derivatives (Pse5Am7Ac8OAc and Pse5Am7Ac8GlnNAc, respectively). Recently, a derivative of pseudaminic acid modified with a di-*O-*methylglyceroyl group was detected in *C. jejuni* NCTC 11168 strain. The gene products required for Pse5Ac7Ac biosynthesis have been characterized, but those genes involved in generating other structures have not. We have demonstrated that the mobility of the NCTC 11168 flagellin protein in SDS-PAGE gels can vary spontaneously and we investigated the role of single nucleotide repeats or homopolymeric-tract-containing genes from the flagellin glycosylation locus in this process. One such gene, Cj1295, was shown to be responsible for structural changes in the flagellin glycoprotein. Mass spectrometry demonstrated that the Cj1295 gene is required for glycosylation with the di-*O-*methylglyceroyl-modified version of pseudaminic acid.

## INTRODUCTION

The Gram-negative, microaerophilic bacterium *Campylobacter jejuni* is one of the most common causes of gastroenteritis in humans. Cells of *C. jejuni* are motile due to the action of single bipolar flagella, and functional flagella are required for both intestinal colonization of animal models (Aguero-Rosenfeld *et al.*, 1990; Nachamkin *et al.*, 1993; Pavlovskis *et al.*, 1991; Wassenaar *et al.*, 1993) and adherence to and invasion of cultured eukaryotic cells (Grant *et al.*, 1993; Wassenaar *et al.*, 1991; Yao *et al.*, 1994). The flagellum filament is formed from polymeric flagellin proteins FlaA and FlaB. These proteins display antigenic variation (Alm *et al.*, 1992; Harris *et al.*, 1987; Logan *et al.*, 2002) and are post-translationally modified by glycosylation (Doig *et al.*, 1996). The glycan component of the flagellin glycoprotein is *O-*linked to multiple (up to 19 in *C. jejuni* strain 81-176) serine/threonine residues in the central domain of the flagellin protein (Thibault *et al.*, 2001). The glycan was initially thought to be *N*-acetyl neuraminic acid (Doig *et al.*, 1996; Guerry *et al.*, 1996) but is now known to be composed of the related sugar 5,7-diacetamido*-*3,5,7,9-tetradeoxy-l-glycero*-*l-mannononulosonic acid or pseudaminic acid (Pse5Ac7Ac) (Thibault *et al.*, 2001). As well as Pse5Ac7Ac, the flagellin from *C. jejuni* 81-176 can also be modified with structural analogues of this basal sugar such as an acetamidino variant (Pse5Am7Ac), as well as variants substituted with acetyl (Pse5Ac7Ac8OAc), propionyl (Pse5Pr7Pr) or *N-*acetyl glutamine structures (Pse5Am7Ac8GlnNAc) (Schirm *et al.*, 2005; Thibault *et al.*, 2001). In *C. jejuni* NCTC 11168, recent structural analyses have identified further novel flagellin modifications consisting of the related sugar legionaminic acid and derivatives, along with di-*O-*methyl glyceric acid derivatives of Pse5Ac7Ac and Pse5Ac7Am (Logan *et al.*, 2009).

The genes involved in *Campylobacter* flagellin glycosylation cluster in a single locus that also contains the tandemly arranged *flaA* and *flaB* genes (Szymanski *et al.*, 2003b; Szymanski & Wren, 2005). The genes encoding the enzymes required for the biosynthesis of cytidine monophosphate-activated pseudaminic acid (CMP-Pse5Ac7Ac) from *N-*acetylglucosamine (GlcNAc) have been identified in both *Campylobacter* and *Helicobacter pylori* (Chou *et al.*, 2005; Creuzenet, 2004; Goon *et al.*, 2003; Ishiyama *et al.*, 2006; Liu & Tanner 2006; Obhi & Creuzenet, 2005; Schirm *et al.*, 2003; Schoenhofen *et al.*, 2006a, b). However, these genes constitute only a subset of those forming the *Campylobacter* flagellin glycosylation locus (Guerry *et al.*, 2006; McNally *et al.*, 2006) and the genes involved in generating the array of structural analogues of pseudaminic acid that are detected on the *Campylobacter* flagellin remain unidentified.

One mechanism for generating structural variation at the bacterial cell surface is phase variation, defined as the random and reversible switching on/off of gene expression. Phase variation in *C. jejuni* and a number of bacterial pathogens such as *Neisseria meningitidis*, *Neisseria gonorrhoeae*, *Haemophilus influenzae* and *H. pylori*, is due to the presence of short, often intragenic, nucleotide repeats (Moxon *et al.*, 2006). Such repeats are relatively unstable due to their inherent susceptibility to a process termed slipped strand mispairing that occurs during DNA replication. The resultant insertion/deletion events produce frame-shifting and populations of cells that differ in their ability to produce full-length proteins. In *Campylobacter coli*, this process mediates a motile to non-motile phenotypic switch due to phase variation of a single nucleotide repeat or homopolymeric tract within the *flhA* gene (Park *et al.*, 2000). The genome sequence of *C. jejuni* NCTC 11168 revealed an abundance of hypervariable homopolymeric tracts of G/C residues (Parkhill *et al.*, 2000). Genes containing such homopolymeric tracts are mostly clustered in three loci involved in biosynthesis of the surface-located structures capsular polysaccharide and lipo-oligosaccharide (LOS), as well as the flagellin glycosylation locus (Parkhill *et al.*, 2000). These homopolymeric tracts have been shown to have a high frequency of insertion/deletion mutations resulting in polymorphic populations of *C. jejuni* cells (Parkhill *et al.*, 2000; Wassenaar *et al.*, 2002). In glycosyltransferase-encoding genes from the LOS biosynthetic locus, phase variation associated with homopolymeric G/C tracts generates LOS structural variation (Gilbert *et al.*, 2002; Linton *et al.*, 2000). Structural analysis of *C. jejuni* capsular polysaccharide identified variation in the glycan component of the glycolipid (Szymanski *et al.*, 2003a), but the role of homopolymeric-tract-containing genes from the capsular biosynthetic locus has not been identified. Translational frameshifting at G/C homopolymeric tracts within *maf* genes from the *C. jejuni* NCTC 11168 flagellin glycosylation locus was shown to partially restore motility in a non-motile *maf* gene mutant (Karlyshev *et al.*, 2002), and more recently, mutation of a homopolymeric-tract-containing *maf* gene resulted in altered flagellin glycosylation (van Alphen *et al.*, 2008). It has also been reported that phase variation of flagellar biosynthesis can be mediated by mutation at single nucleotide repeats within the genes encoding the FlgSR two-component system (Hendrixson, 2006, 2008).

It has been known for some time that *Campylobacter* flagellin proteins can undergo spontaneous antigenic variation (Harris *et al.*, 1987). More recently, considerable structural variation of the flagellin glycan has been demonstrated; however, the genetic basis of this structural variation has not been determined. In this report, we demonstrate that the homopolymeric-tract-containing Cj1295 gene, encoding a protein of unknown function, is responsible for generating a defined structural variant of the glycan component of the *C. jejuni* flagellin glycoprotein.

## METHODS

### Bacterial strains and growth conditions.

All *C. jejuni* strains were grown at 37 °C on 7 % horse blood (TCS Biosciences) agar supplemented, as required, with kanamycin (50 μg ml^−1^) and chloramphenicol (20 μg ml^−1^) in a microaerobic chamber (Don Whitley) with an atmosphere of 10 % carbon dioxide, 5 % oxygen and 85 % nitrogen. Motility of *C. jejuni* strains was analysed by inoculating 0.4 % agar Mueller–Hinton plates. *Escherichia coli* strains were grown in Luria–Bertani (LB) broth or on LB agar at 37 °C supplemented, as required, with ampicillin (100 μg ml^−1^), kanamycin (50 μg ml^−1^) and chloramphenicol (20 μg ml^−1^).

### Insertional mutagenesis of the *C. jejuni* NCTC 11168 Cj1295 gene.

A 1308 bp PCR product was amplified from *C. jejuni* NCTC 11168 genomic DNA with primers 1295mutf (5′-GTG GAT AAG CTA GAC TTT AGA-3′) and 1295mutr (5′-TCA TTT TAC CAG TCC ATA AAA-3′) and cloned into plasmid pGEM-T Easy (Invitrogen). The resultant plasmid (p1295) was cut at the single *Bcl*I site located at nt 789–794 inclusive of the 1308 bp insert generated by PCR; a kanamycin cassette (Trieu-Cuot *et al.*, 1985) with *Bam*HI termini was ligated into this site to generate plasmid p1295 : : aphA. Following transformation into *E. coli* TOP 10 cells (Invitrogen), transformants were selected on kanamycin-containing media and restriction digestion was used to identify plasmids with the kanamycin cassette in the same orientation as the Cj1295 gene. This plasmid was electroporated into *C. jejuni* NCTC 11168 using standard methods (van Vliet *et al.*, 1998) and transformants were verified by PCR.

### Complementation of the Cj1295 gene knockout mutant.

In order to complement the Cj1295 knockout mutant, we integrated a functional copy of the Cj1295 gene onto the chromosome. To minimize disruption of other genes we targeted a region annotated as a pseudogene (Cj0223) in the *C. jejuni* NCTC 11168 genome sequence and a vector designed to integrate functional genes onto the chromosome was designed. This was based on a 2179 bp fragment corresponding to nt 205297–207475 inclusive of the *C. jejuni* NCTC 11168 genome sequence, cloned into pUC18. In order to facilitate insertion of genes into the centre of this region, a *Spe*I restriction site was created using site-directed mutagenesis. The primer 5′-GGT GTA GTA AGT ACT AGT AAT TGT AAT GTC C-3′ and its exact complement were employed to generate the *Spe*I site (underlined).

In parallel, a 1411 bp PCR product was amplified from *C. jejuni* NCTC 11168 genomic DNA using primers 1295F2 (5′-GCT GAA CTT AGT ATA CCT TGT-3′) and 1295mutr (see above) with *Pfu* proof-reading polymerase (Stratagene). This amplified DNA from 103 bp upstream of the putative start codon of the Cj1295 gene through to the putative stop codon. This PCR product was A-tailed with *Taq* polymerase (Bioline) and cloned into pGEM-T Easy (Invitrogen). The single nucleotide repeat of G residues was then altered using site-directed mutagenesis employing a pair of complementary primers: 1295mutf (5′-TAA ATA AAA CTC TGG GAG GGG GTA TAC TC-3′) and the exact complement 1295mutr.

A chloramphenicol resistance cassette was amplified from plasmid pAV35 (van Vliet *et al.*, 1998) using primers catF*Aat*II*Spe*I (5′-TGA CTG GAC GTC ACT AGT TGC TCG GCG GTG TTC CTT TCA AG-3′) and catR*Sac*II (5′-TCA CTG CCG CGG TTA TTT ATT CAG CAA GTC TTG TAA-3′), digested with *Aat*II and *Sac*II and cloned into *Aat*II/*Sac*II-digested plasmid p1295QC. The chloramphenicol resistance cassette and full-length Cj1295 gene with an interrupted G/C tract were cut out from this plasmid with *Spe*I and ligated into the *Spe*I site of the pseudogene-containing pUC18 based plasmid (see above) to create plasmid pCj1295comp.

Following sequence verification of the Cj1295 gene, this construct was electroporated into *C. jejuni* NCTC 11168 and chloramphenicol-resistant colonies were isolated. Genomic DNA was extracted from a chloramphenicol-resistant strain and checked for insertion of the full-length Cj1295 gene with interrupted G/C tract along with the chloramphenicol resistance cassette into the pseudogene Cj0223 by PCR. Genomic DNA from this strain was used to naturally transform strain *C. jejuni* NCTC 11168 Cj1295 : : aphA to chloramphenicol resistance using established methods (van Vliet *et al.*, 1998). Kanamycin- and chloramphenicol-resistant colonies were isolated and the site of insertion of the functional Cj1295 gene with interrupted G/C tract was verified by PCR and sequencing.

### Analysis of flagellin mobility by protein electrophoresis and Western blotting.

Whole-cell protein extracts were electrophoresed through 12 % Bis/Tris polyacrylamide gels (Invitrogen), according to the manufacturer's instructions. Gels were either stained with colloidal Coomassie stain (Pierce) or electroblotted to PVDF membranes. Flagellin proteins were visualized using a polyclonal anti-flagellin antibody (kindly supplied by Dr Shaun Cawthraw and Professor Diane Newell) and anti-rabbit IgG peroxidase-coupled secondary antibody (Sigma) with DAB staining (Vector).

### Partial purification of flagellin protein and analysis by mass spectrometry.

Flagellin protein was purified using the method described by Power *et al.* (1994). Purified flagellin was separated by SDS-PAGE and stained using Novex colloidal blue reagent (Invitrogen) and the desired protein was excised, lyophilized and digested with trypsin (E.C.3.4.21.4, Promega) overnight. Peptides were then extracted from gel pieces and analysed by nano-liquid chromatography/electrospray ionization (LC-ES)–MS/MS using a reverse-phase nano-HPLC system (Dionex) connected to a quadrupole time-of-flight mass spectrometer (Q-STAR Pulsar I, MDS Sciex). The digests were separated by a binary nano-HPLC gradient generated by an Ultimate pump fitted with a Famos autosampler and a Switchos microcolumn switching module (LC Packings). An analytical C_18_ nanocapillary (75 μm inside diameter×15 cm; PepMap) and a micro precolumn C_18_ cartridge were employed for online peptide separation. The digest was first loaded onto the precolumn and eluted with 0.1 % formic acid (Sigma) in water (HPLC grade; Purite) for 4 min. The eluant was then transferred onto an analytical C_18_ nanocapillary HPLC column and eluted at a flow rate of 150 nl min^−1^. The following gradient was employed: 99 % solvent A from 0 to 5 min, 99–90 % A from 5 to 10 min, 90–60 % A from 10 to 70 min, 60–50 % A from 70 to 71 min, 50–5 % A from 71 to 75 min, 5 % A from 75 to 85 min, 5–95 % A from 85 to 86 min, and 95 % A from 86 to 90 min. Solvent A consisted of 0.05 % (v/v) formic acid in a 95 : 5 (v/v) water/acetonitrile mixture, whilst solvent B consisted of 0.04 % formic acid in a 95 : 5 (v/v) acetonitrile/water mixture. Data acquisition was performed using Analyst QS software with an automatic information-dependent-acquisition function.

## RESULTS

### Instability of homopolymeric-tract-containing genes is associated with altered flagellin mobility

**Fig. 1.**
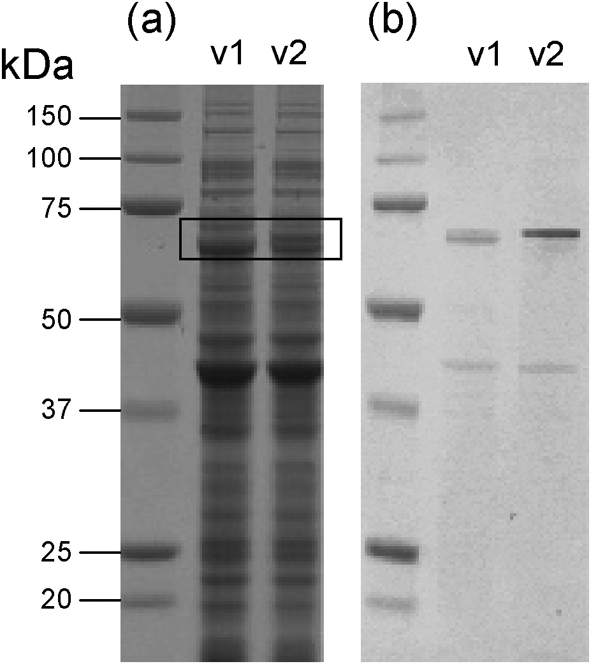
Altered mobility of flagellin protein from motile variants of *C. jejuni* NCTC 11168. (a) Whole-cell protein profiles of variants v1 and v2 of *C. jejuni* NCTC 11168. The boxed region outlines a region of the gel where there is apparent variation. (b) Western blot (a) probed with anti-flagellin antiserum. A clear flagellin protein mobility shift is evident when comparing v1 and v2. The faint band observed between 37 and 50 kDa is due to non-specific binding of antibody to the major outer-membrane protein.

Whilst investigating flagella-mediated motility in *C. jejuni* NCTC 11168, two single colony-derived motile variants with a minor difference in whole-cell protein profile between the 50 kDa and 75 kDa markers were obtained by chance (boxed in Fig. 1a). In order to determine whether this was due to heterogeneity of the approximately 60 kDa flagellin protein, Western blotting was carried out using a flagellin-specific antiserum (Fig. 1b). This clearly demonstrated that the flagellin proteins from the two variants, termed v1 and v2, had different electrophoretic mobilities.

We hypothesized that these flagellin mobility variants arose due to changes in hypervariable homopolymeric tracts, more specifically 12 homopolymeric G/C tracts from those identified by Parkhill *et al.* (2000) that are potentially involved in flagellin modification (Table 1). Within the *C. jejuni* NCTC 11168 flagellin glycosylation locus, there are ten homopolymeric tracts of G/C residues, nine of which are intragenically located, indicating potentially phase variable genes, along with a single intergenic homopolymeric tract of G residues located just upstream of the Cj1321 gene. Two further homopolymeric G/C tracts located outside of the flagellin glycosylation locus were also included as they were located within genes (Cj0617 and Cj0170/1) that had significant levels of sequence similarity to homopolymeric tract-containing genes from the flagellin glycosylation locus (Table 1). The repeat containing regions from each of the 12 homopolymeric tract-containing genes associated with flagellin glycosylation were amplified by PCR and the tract was sequenced. Comparison of the on/off status of the corresponding genes identified three flagellin glycosylation-associated genes that differed in their translational on/off status between the flagellin mobility variants NCTC 11168 v1 and v2. Two of these, the adjacent Cj1305 and Cj1306 genes, belong to the Cj0617 paralogous gene family encoding putative proteins of unknown function. In the v1 variant, the Cj1305 gene is in-frame and Cj1306 is out-of-frame, whilst in the v2 variant, the Cj1305 gene is out-of-frame and Cj1306 is in-frame. The third gene that differed in translational on/off status between flagellin mobility variants NCTC 11168 v1 and v2 is coding sequence Cj1295. In the v1 variant, Cj1295 is out-of-frame with eight G residues, whilst in the v2 variant, the Cj1295 gene is in-frame with nine G residues (Table 1). Given the complexity of the Cj0617 paralogous gene family and that Cj1305 and Cj1306 both differed in translational status between v1 and v2, we decided to investigate the role of the homopolymeric-tract-containing Cj1295 gene in flagellin modification.

### Analysis of the *C. jejuni* Cj1295 gene

The *C. jejuni* NCTC 11168 Cj1295 predicted coding sequence encodes a putative protein of 435 aa (49.9 kDa) that lacks both a predicted signal peptide sequence and transmembrane regions. This putative protein belongs to the Protein Information Resource PIRSF015244 family with the tentative function of ‘polysaccharide biosynthesis protein with an aminopeptidase-like domain’. Members of this protein family are found in phylogenetically diverse bacterial species, including *Frankia* and *Streptomyces*, from the phylum *Actinobacteria*; *Bradyrhizobium* and *Brucella*, from the *Alphaproteobacteria*; *Hydrogenivirga* from the *Aquificae*; *C. jejuni*, *C. coli*, *Campylobacter lari*, *Campylobacter fetus* and *Campylobacter upsaliensis* from the *Delta*-/*Epsilonproteobacteria*; *Clostridium acetobutylicum* from the phylum *Firmicutes*; and *Aeromonas* from the *Gammaproteobacteria*. In the majority of species, the coding sequences encoding Cj1295-like proteins are located within clusters of putative genes involved in sugar biosynthesis/modification or sugar transferases. A number of these clusters also contain genes annotated as sialic acid synthases. For *Campylobacter* spp. and *C. acetobutylicum*, Cj1295 homologue-containing gene clusters encode proteins involved in sugar biosynthesis/modification and flagellum biosynthesis.

In *C. jejuni*, the Cj1295 coding sequence is part of a large cluster of genes, a number of which encode enzymes known to be involved in glycosylation of the flagellin protein. This locus varies in gene content among *C. jejuni* strains; however, Cj1295-like coding sequences are present in all ten *C. jejuni* strains for which sequence data are available, along with *C. coli*, *C. lari*, *C. fetus* and *C. upsaliensis*. Comparison of *C. jejuni* Cj1295-like coding sequences from different strains reveals high levels of nucleotide sequence identity apart from at the homopolymeric tract of G/C residues located in the 5′ region of the gene.

### Construction of ‘knock-out’ and ‘knock-in’ Cj1295 genes

In order to investigate Cj1295 gene product function, an insertional knockout mutant was created in *C. jejuni* NCTC 11168 to create *C. jejuni* NCTC 11168 Cj1295 : : aphA. For comparison, a Cj1295 gene was constructed with an interrupted homopolymeric tract. Full-length Cj1295 was cloned and site-directed mutagenesis was employed to alter the homopolymeric tract of G residues so that (i) the gene was in-frame, (ii) the run of G residues was interrupted and (iii) there was no change to the amino acid sequence. This was achieved by inserting an adenosine (A) residue to replace a G residue so that the resulting gene contained a region consisting of three G residues followed by a single A residue followed by five G residues rather than a continuous run of nine G residues.

In order to construct a *C. jejuni* strain expressing a Cj1295 gene with an interrupted homopolymeric tract, the engineered variant was recombined onto the chromosome at a site distant from the flagellin glycosylation locus. To minimize possible secondary effects of gene insertion, the site chosen was within a pseudogene (annotated as Cj0223). Expression of the Cj1295 gene with an interrupted homopolymeric tract was under the control of the promoter associated with a chloramphenicol resistance gene placed immediately upstream and in the same transcriptional orientation. Insertion of the adjacent chloramphenicol resistance and Cj1295 genes within the Cj0223 pseudogene on the *C. jejuni* chromosome was verified by PCR (data not shown).

### Cj1295 gene function is associated with reduced electrophoretic mobility of the flagellin glycoprotein

**Fig. 2.**
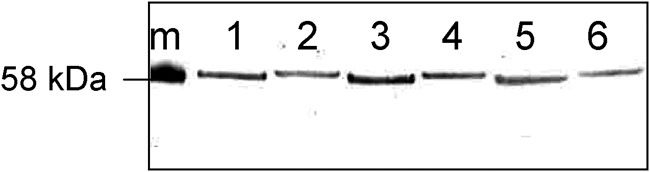
Flagellin mobility shift due to Cj1295 gene function. Three kanamycin-resistant colonies with non-functional Cj1295 genes (lanes 1, 3 and 5) and three kanamycin- and chloramphenicol-resistant colonies with functional Cj1295 genes (lanes 2, 4 and 6) were analysed by Western blotting with an anti-flagellin antibody. Lane m, molecular mass marker.

The possibility that the instability of other homopolymeric-tract-containing genes might contribute to any phenotypic changes observed when studying Cj1295 gene function necessitated the following approach. Genomic DNA from NCTC 11168 with a Cj1295 gene with an interrupted homopolymeric tract inserted into the pseudogene Cj0223 (see above), was used to naturally transform *C. jejuni* NCTC 11168 Cj1295 : : aphA. From the transformation mixture, both singly (kanamycin) and doubly (kanamycin and chloramphenicol) resistant colonies were isolated. The kanamycin-resistant colonies lack a functional Cj1295 gene whilst the doubly kanamycin- and chloramphenicol-resistant colonies contain a functional Cj1295 gene. The mobility of the flagellin protein was determined directly from three kanamycin-resistant and three kanamycin/chloramphenicol-resistant single colonies. In agreement with data obtained from variants v1 and v2 (see above), the flagellin glycoprotein from the kanamycin-resistant colonies lacking a functional Cj1295 gene had decreased mobility in SDS-PAGE gels compared with flagellin from the kanamycin/chloramphenicol-resistant colonies containing a functional Cj1295 gene (Fig. 2). This experiment was repeated three times and in each case, a similar shift in flagellin mobility was observed. These data indicate that Cj1295 function is involved in modification of the flagellin protein and that inherent instability of the homopolymeric tract would result in distinct populations of *C. jejuni* NCTC 11168 with structurally variant flagellin glycoprotein. Despite these variant flagellins, both strains were fully motile (as assessed by point-inoculating reduced agar plates), both were flagellated (as assessed by electron microscopy) and there was no difference in their capacity to autoagglutinate (data not shown).

### Cj1295 gene function is associated with the di-*O*-methyl-glyceric acid modification of pseudaminic acid

Flagellin proteins from *C. jejuni* NCTC 11168 1295 : : aphA and the complemented strain, were partially purified by SDS-PAGE and analysed by mass spectrometry. Gel pieces corresponding to flagellin were subjected to in*-*gel trypsin digestion and the resulting peptides were eluted and analysed by LC-ES-MS/MS. The data were analysed for the presence of oxonium ions at *m*/*z* 317 and 316, which correspond to the flagellin glycan Pse5Ac7Ac and its acetamidino derivative Pse5Am7Ac, respectively, previously demonstrated to modify the flagellin of *C. jejuni* 81-176 (Thibault *et al.*, 2001). Additionally, it has recently been reported that the flagellin of *C. jejuni* NCTC 11168 is modified with a di-*O-*methyl-glyceric acid derivative of pseudaminic acid (Logan *et al.*, 2009). Accordingly, the data were also analysed for the presence of oxonium ions at *m*/*z* 391 and 390, which are indicative of the presence of this pseudaminic acid derivative on flagellin glycopeptides.

**Fig. 3.**
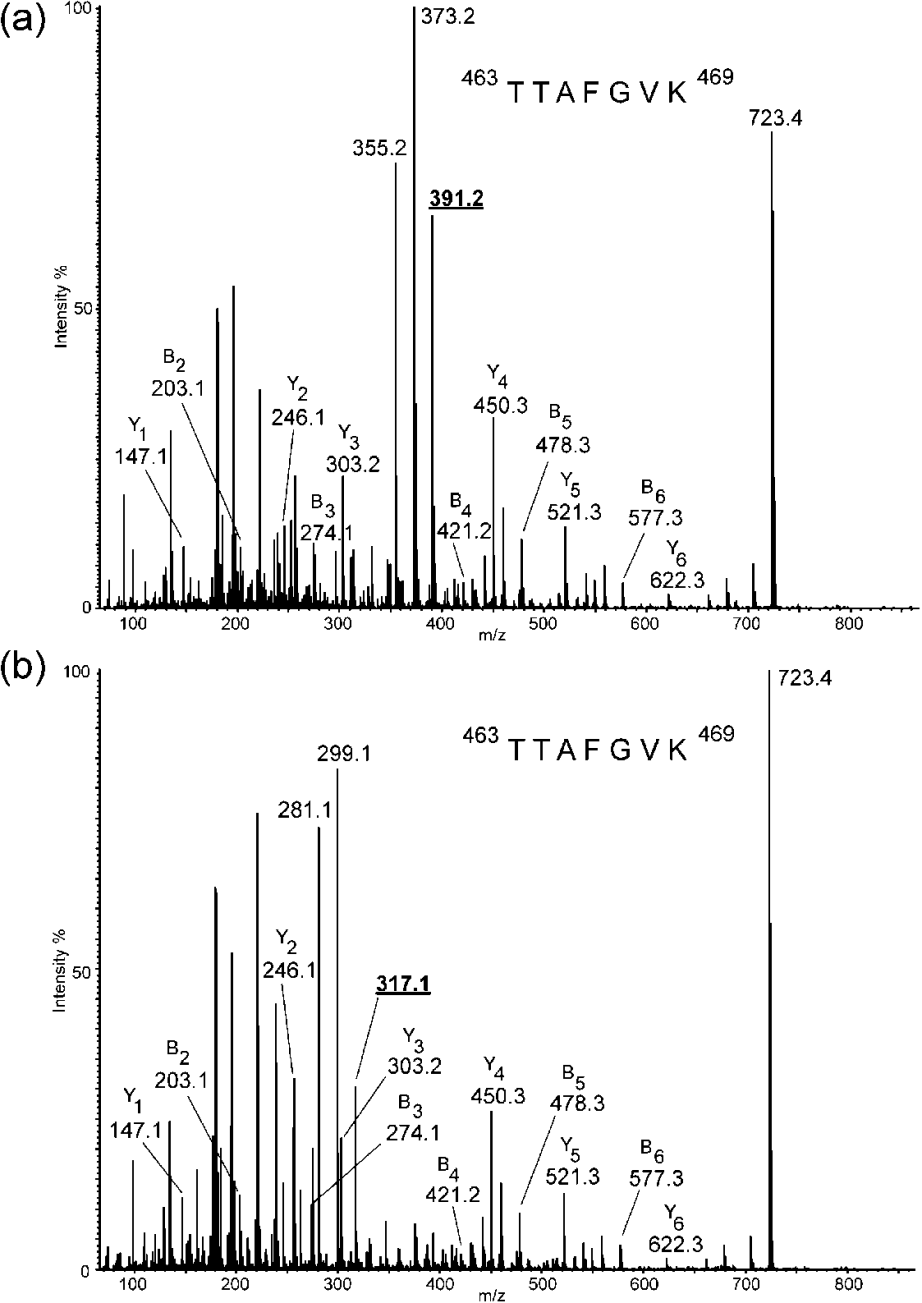
NanoLC-ES-MS/MS analysis of the tryptic digest of *C. jejuni* 11168 flagellin. The spectra show the glycopeptide ^463^TTAFGVK^469^ modified with a single glycan. (a) The doubly charged ion at *m*/*z* 557.3 derived from the NCTC 11168 Cj1295 : : aphA cmCj1295 strain expressing full-length Cj1295 with an interrupted homopolymeric tract. The characteristic oxonium ion at *m*/*z* 391 for the dimethylglyceric acid analogue of pseudaminic acid is underlined. (b) The doubly charged ion at *m*/*z* 520.3 derived from the NCTC 11168 Cj1295 : : aphA strain that does not express the Cj1295 gene product. The characteristic oxonium ion at *m*/*z* 317 for pseudaminic acid is underlined.

The data from the LC-ES-MS/MS analysis of the flagellin from *C. jejuni* NCTC 11168 Cj1295 : : aphA cmCj1295 strain indicated that the most abundant modification found on the flagellin glycopeptides was the dimethylglyceric acid derivative of Pse5Ac7Ac (oxonium ion at *m*/*z* 391; Fig. 3a and Table 2). Glycopeptides were also detected that were modified with its related acetamidino derivative (oxonium ion at *m*/*z* 390) and with Pse5Ac7Ac, respectively (Table 2).

In contrast, the LC-ES-MS/MS analysis following trypsin digestion of the flagellin from NCTC 11168 Cj1295 : : aphA strain indicated the distinct absence of glycopeptides modified with dimethylglyceric acid pseudaminic acid derivative. The glycopeptides observed were modified with Pse5Ac7Ac or its acetamidino derivative (oxonium ions at *m*/*z* 317 or 316, respectively; Fig. 3b and Table 2). The increased mass of the dimethylglyceric acid derivatives of pseudaminic acid relative to Pse5Ac7Ac/Pse5Ac7Am correlates with the decreased mobility in SDS-PAGE gels of the flagellin protein modified with this sugar given that there are multiple sites of glycosylation on the flagellin protein.

## DISCUSSION

The identification of a large number of homopolymeric tracts of G/C residues, many of which were shown to be unstable, within genes from chromosomal loci involved in biosynthesis of capsular polysaccharide, LOS and the flagellin glycoprotein was the most significant finding of the *C. jejuni* NCTC 11168 genome sequencing project (Parkhill *et al.*, 2000). The resultant on/off switching in the translational status of homopolymeric-tract-containing genes is thought to mediate generation of structural diversity of the bacterial cell surface. Indeed, the presence of many homopolymeric-tract-containing genes in the flagellin glycosylation locus is congruent with the observed structural heterogeneity of the glycan component of the flagellin glycoprotein (Logan *et al.*, 2009; Schirm *et al.*, 2005; Thibault *et al.*, 2001). However, despite considerable progress in defining the genes from this locus that encode the enzymes required for biosynthesis of Pse5Ac7Ac, the most commonly detected flagellin modification (Chou *et al.*, 2005; Creuzenet, 2004; Goon *et al.*, 2003; Ishiyama *et al.*, 2006; Liu & Tanner 2006; Obhi & Creuzenet 2005; Schirm *et al.*, 2003; Schoenhofen *et al.*, 2006b), little progress has been made in determining how the observed structural variants of Pse5Ac7Ac are generated and what role homopolymeric tract-containing genes may have. We have tested the hypothesis that homopolymeric-tract-containing genes from the flagellin glycosylation locus are involved in generating flagellin glycan diversity by identifying spontaneously generated intra-strain variation in mobility of the *C. jejuni* NCTC 11168 flagellin glycoprotein and associating this with differences in the translational status of homopolymeric-tract-containing genes Cj1305, Cj1306 and Cj1295 from the flagellin glycosylation locus. We have gone on to define more precisely the role of Cj1295 in generating variant flagellins of differing mobility and demonstrated that this is due to altered flagellin glycan structure. This study is therefore an important first step towards defining the components of the flagellin glycosylation machinery that are involved in generating structural diversity in the *Campylobacter* flagellin glycan.

The approach employed to investigate Cj1295 gene function involved construction of a *C. jejuni* NCTC 11168 Cj1295 ‘knock-out’ strain by standard insertional knockout mutagenesis along with a so-called ‘knock-in’ strain. The latter was generated by interrupting the homopolymeric tract of G residues and then introducing this gene into the genome of the ‘knock-out’ strain within a pseudogene (see Methods). This strategy ensured direct comparison of isogenic strains differing only in their capacity to produce a functional Cj1295 gene product. When flagellin proteins from these strains were compared, there was a clear difference in their mobility in SDS-PAGE gels so that a functional Cj1295 gene was associated with a flagellin glycoprotein of reduced mobility. When conducting such experiments, it is important that the contribution of other unstable homopolymeric-tract-containing genes from the flagellin glycosylation locus is considered, as their spontaneous frame-shifting may also lead to modification of the flagellin glycan with consequent mobility shifting. Therefore, we repeated the transformation experiments three times and analysed the flagellin mobility of multiple single colonies directly from the primary transformation plates, i.e. without subsequent subculture. In all cases, a consistent flagellin mobility shift was observed. We further investigated the cause of this mobility shift by mass spectrometry, showing that the Cj1295 gene function was associated with the presence of a di-*O-*methyl glyceric acid-derivative of pseudaminic acid (Logan *et al.*, 2009). The full complement of NCTC 11168 flagellin glycosylation sites has not yet been identified, but our data (Table 2) suggest that the majority are modified with the di-*O-*methyl glyceric acid derivative of pseudaminic acid when the Cj1295 gene is functional.

The Cj1295 gene is located at one end of the large cluster of genes known as the flagellin glycosylation locus. A number of genes from this locus (including Cj1293 and Cj1294 located immediately upstream of Cj1295) are involved in the biosynthesis of pseudaminic acid (Goon *et al.*, 2003; Obhi & Creuzenet, 2005), the principal glycan component of the flagellin glycoprotein. Indeed, all genes necessary for the biosynthesis of cytidine monophosphate-activated pseudaminic acid (CMP-Pse5Ac7Ac) from GlcNAc have been identified (Chou *et al.*, 2005; Creuzenet, 2004; Goon *et al.*, 2003; Ishiyama *et al.*, 2006; Liu & Tanner 2006; Obhi & Creuzenet, 2005; Schirm *et al.*, 2003; Schoenhofen *et al.*, 2006a, b), and the corresponding gene products are capable of synthesizing CMP-Pse5Ac7Ac from GlcNAc *in vitro* (Schoenhofen *et al.*, 2006a). However, analysis of the *Campylobacter* flagellin glycan from strains 81-176 and NCTC 11168 has clearly shown that, as well as Pse5Ac7Ac, a number of structurally related analogues are present (Logan *et al.*, 2009; Schirm *et al.*, 2005; Thibault *et al.*, 2001). The predicted amino acid sequence of the Cj1295 gene product contains an aminopeptidase-like domain, yet the Cj1295 gene product activity is associated with changes not to the flagellin polypeptide but to the flagellin glycan, more specifically, presence of a pseudaminic acid-like sugar of 389–390 Da. In a very recent report, this modification in NCTC 11168 flagellin was identified by mass spectrometry, NMR and metabolomics as CMP-7-acetamido-5-(2,3-di-*O-*methylglyceroyl)amino-3,5,7,9-tetradeoxy-l-glycero-*α*-l-manno-nonulosonic acid (Logan *et al.*, 2009). We hypothesize that the Cj1295 gene product is directly involved in generating this modification. Our current working hypothesis is, that in cells where the Cj1295 gene product is produced, the protein acts to cleave the acetamido group on carbon 5 of the basal Pse5Ac7Ac/Pse5Ac7Am sugar. The presence of an aminopeptidase-like domain in Cj1295 suggests that the covalent bond linking the amide group, which resembles a peptide bond, may be the cleavage site enabling substitution of the acetamido group with a methylglyceroyl group. Knockout mutation of genes involved in flagellin glycosylation that result in altered flagellin charge train patterns have also been shown to alter autoagglutination properties of the corresponding *C. jejuni* cells (Guerry *et al.*, 2006; van Alphen *et al.*, 2008). We observed no change in autoagglutination associated with the presence or absence of a functional Cj1295 gene, presumably because the associated glycan variation did not result in altered charge.

The Cj1295 gene is one of ten homopolymeric-tract-containing genes found within the flagellin glycosylation locus of strain NCTC 11168. Of particular interest are the Cj0617 gene family in which all five members (four located in the flagellin glycosylation locus) contain homopolymeric tracts. Also of interest are a second paralogous gene family known as the motility accessory factor or *maf* genes (Karlyshev *et al.*, 2002). Of the seven *maf* genes present in the *C. jejuni* NCTC 11168 flagellin glycosylation locus, two contain homopolymeric tracts. Both the Cj0617 and *maf* gene family, along with other homopolymeric-tract-containing genes of the flagellin glycosylation locus are likely to contribute to generating diversity in the flagellin glycan, although their specific contributions will require further detailed study. Comparison of flagellin glycosylation loci from different strains of *C. jejuni* reveals considerable diversity in gene content, but the Cj1295 gene is present in all strains for which genome sequence data are available and in each case contains a homopolymeric tract.

Phase variable expression of short sequence repeat-containing genes encoding glycosyltransferases leads to variant glycans of meningococcal and gonococcal pilin glycoproteins (Aas *et al.*, 2007; Banerjee *et al.*, 2002; Power *et al.*, 2003). In a similar manner, the Cj1295 homopolymeric-tract-containing gene generates variant flagellin glycosylation and it is interesting to speculate on such diversity in *C. jejuni*. One possible explanation is that during colonization, the random generation of structural diversity in the flagellin glycan is important in evading host immune responses. The flagellin protein is an immunodominant antigen in *C. jejuni*-colonized chickens (Cawthraw *et al.*, 1994) and in chickens immunized with *Campylobacter* antigens (Widders *et al.*, 1998). Flagellin is also recognized by antibodies from the sera of humans infected with *C. jejuni* (Cawthraw *et al.*, 2002; Nachamkin & Hart, 1985; Wenman *et al.*, 1985). Alternatively, the generation of such diversity may be involved in preventing the binding of flagellatropic bacteriophages (Coward *et al.*, 2006).

## Figures and Tables

**Table 1.** Sequence of homopolymeric tracts associated with the *C. jejuni* NCTC 11168 flagellin glycosylation locus in flagellin mobility variants

**CDS**	**Putative function**	Length of single nucleotide repeat (bp) and consequent translational status*	
		**Variant 1**	**Variant 2**
Cj1295	Aminopeptidase	8 (−)	9 (+)
Cj1296/7	*N-*Acetyltransferase	8 (−)	8 (−)
Cj1305	Unknown (Cj0617 family)	9 (+)	10 (−)
Cj1306	Unknown (Cj0617 family)	8 (−)	9 (+)
Cj1310	Unknown (Cj0617 family)	9 (+)	9 (+)
Cj1342	Unknown (Cj0617 family)	9 (+)	9 (+)
Cj1325/6	Unknown	10 (−)	10 (−)
Cj0617	Unknown (Cj0617 family)	10 (+)	10 (+)
Upstream of Cj1321	Transferase	10	10
Cj1317/8	Unknown (Cj1318 family)	9 (−)	9 (−)
Cj1334	Unknown (Cj1318 family)	9 (−)	9 (−)
Cj0170/1	Unknown	10 (−)	9 (−)

*+, In-frame; −, out-of-frame.

**Table 2.** LC-ES-MS/MS analysis of flagellin-derived tryptic glycopeptides from the NCTC 11168 Cj1295 : : aphA cmCj1295 strain expressing a full-length Cj1295 gene with an interrupted homopolymeric tract (Cj1295+) and the NCTC 11168 Cj1295 : : aphA strain (Cj1295−)

**Peptide**	**Observed glycopeptide signal (*m*/*z*)**	**Glycan oxonium ion**	**Deduced [M+H]^+^**	**Peptide sequence**	**Modification**	**Flagellin**
**Cj1295+**						
T463-469	557.3 [M+2H]^2+^	391	1113.5	TTAFGVK	Pse5dimethylglycerate7Ac	FlaA, FlaB
T179-190	843.4 [M+2H]^2+^	391	1685.8	ISSSGEVQFTLK	Pse5dimethylglycerate7Ac	FlaB
T179-190	850.4 [M+2H]^2+^	391	1699.8	ISTSGEVQFTLK	Pse5dimethylglycerate7Ac	FlaA
T203-222	1167.6 [M+2H]^2+^	391	2334.2	VVISTSVGTGLGALADEINK	Pse5dimethylglycerate7Ac	FlaA
	1130.6 [M+2H]^2+^	317	2260.2		Pse5Ac7Ac	
T203-222	783.4 [M+3H]^3+^; 1174.3 [M+2H]^2+^	391	2348.2	VVISTSVGTGLGALAEEINK	Pse5dimethylglycerate7Ac	FlaB
T338-365	1202.2 [M+3H]^3+^	390	3604.7	DILISGSNLSSAGFGATQFISQASVSLR	Pse5dimethylglycerate7Am ×2	FlaA, FlaB
**Cj1295−**						
T463-469	520.3 [M+2H]^2+^	317	1039.4	TTAFGVK	Pse5Ac7Ac	FlaA, FlaB
T179-190	806.4 [M+2H]^2+^	317	1611.7	ISSSGEVQFTLK	Pse5Ac7Ac	FlaB
T179-190	813.4 [M+2H]^2+^	317	1625.7	ISTSGEVQFTLK	Pse5Ac7Ac	FlaA
T203-222	1130.6 [M+2H]^2+^	317	2260.1	VVISTSVGTGLGALADEINK	Pse5Ac7Ac	FlaA
T203-222	1137.6 [M+2H]^2+^	317	2274.1	VVISTSVGTGLGALAEEINK	Pse5Ac7Ac	FlaB
T338-365	1153.6 [M+3H]^3+^	316	3456.7	DILISGSNLSSAGFGATQFISQASVSLR	Pse5Ac7Am x 2	FlaA, FlaB

## Abbreviations

- GlcNAc, *N-*acetylglucosamine
- LB, Luria–Bertani
- LC-ES, liquid chromatography/electrospray ionization
- LOS, lipo-oligosaccharide
